# PD-1 and PD-L1 expression on PBMC subsets in normal individuals and cancer patients

**DOI:** 10.1186/2051-1426-2-S3-P152

**Published:** 2014-11-06

**Authors:** Lauren Lepone, Renee N Donahue, Italia Grenga, James L Gulley, Christopher R Heery, Ravi A Madan, Jeffrey Schlom, Benedetto Farsaci

**Affiliations:** 1 Laboratory of Tumor Immunology and Biology, CCR, NCI, NIH, Bethesda, MD, USA; 2 CCR, NCI, NIH, Bethesda, MD, USA; 3 Genitourinary Malignancies Branch, CCR, NCI, NIH, Bethesda, MD, USA

## Purpose

Immunotherapies aiming to interfere with the immune checkpoint molecule PD-1 (programmed death-1) and its ligand PD-L1 are currently being investigated in several clinical trials to treat cancer patients. The PD-1 pathway is one of the ways cancer cells evade immune-mediated killing. As little is known about the expression of PD-1 and PD-L1 in cancer patients compared to normal individuals, the aim of this study was to assess PBMC subsets for expression of these markers.

## Methods

**Figure 1:**
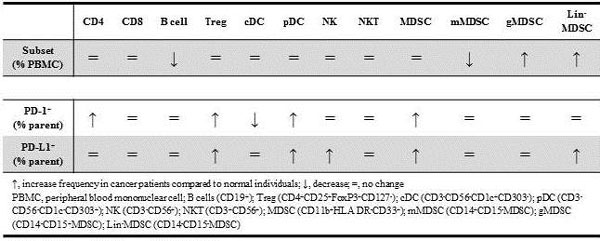
**Differences in PBMC subsets and PD-1 and PD-L1 expression in cancer patients at baseline and normal individuals**.

Twelve immune cell subsets were analyzed by flow-cytometry in 22 cancer patients and 16 normal individuals. The cancer patients consisted of 1 anal, 2 breast, 4 colon, 1 esophageal, 2 mesothelioma, 1 neuroendocrine, 1 non-small cell lung, 1 ovarian, 5 pancreatic, 3 renal cell and 1 squamous cell tracheal cancer patients. The subsets analyzed were CD4 and CD8 T cells, B cells, conventional dendritic cells (cDC), plasmacytoid DC (pDC), natural killer cells (NK), natural killer T cells (NKT), myeloid derived suppressor cell (MDSC), monocytic MDSC (mMDSC), granulocytic MDSC (gMDSC), and lineage-negative MDSC (Lin-MDSC). We also analyzed surface expression of PD-1 (clone MIH4) and PDL-1 (clone MIH1). See Figure 1.

## Results

Compared to normal subjects, cancer patients had some PBMC subsets with changes in frequency but no differences in PD-1 and PD-L1 expression (i.e., B cells, mMDSCs, and gMDSCs). Other subsets showed changes in PD-1 and PD-L1 expression without differences in the frequency of the subset (i.e., CD4, Tregs, cDCs, pDCs, NK, and MDSCs). Lin-MDSCs presented at a higher frequency and greater PD-L1 positivity.

## Conclusions

Understanding the differences of PBMC immune subsets between normal subjects and cancer patients, and the surface expression of PD-1 and PD-L1, can provide insights as to which immune subsets can be targeted by therapies aimed at interfering with the PD-1 pathway in cancer patients.

